# The Capsid Protein of Rubella Virus Antagonizes RNA Interference in Mammalian Cells

**DOI:** 10.3390/v13020154

**Published:** 2021-01-21

**Authors:** Jiuyue Xu, Jing Kong, Bao Lyu, Xiaotong Wang, Qi Qian, Xi Zhou, Yang Qiu

**Affiliations:** 1 State Key Laboratory of Virology, Wuhan Institute of Virology, Center for Biosafety Mega-Science, Chinese Academy of Sciences (CAS), Wuhan 430071, Hubei, China; wizardsugar@163.com (J.X.); 18844545827@163.com (J.K.); baolyu@163.com (B.L.); wangxt19960601@163.com (X.W.); qianqi91@163.com (Q.Q.); 2 University of Chinese Academy of Sciences, Beijing 100049, China

**Keywords:** antiviral RNAi, capsid, rubella virus, viral suppressors of RNAi

## Abstract

Rubella virus (RuV) is the infectious agent of a series of birth defect diseases termed congenital rubella syndrome, which is a major public health concern all around the world. RNA interference (RNAi) is a crucial antiviral defense mechanism in eukaryotes, and numerous viruses have been found to encode viral suppressors of RNAi (VSRs) to evade antiviral RNAi response. However, there is little knowledge about whether and how RuV antagonizes RNAi. In this study, we identified that the RuV capsid protein is a potent VSR that can efficiently suppress shRNA- and siRNA-induced RNAi in mammalian cells. Moreover, the VSR activity of the RuV capsid is dependent on its dimerization and double-stranded RNA (dsRNA)-binding activity. In addition, ectopic expression of the RuV capsid can effectively rescue the replication defect of a VSR-deficient virus or replicon, implying that the RuV capsid can act as a VSR in the context of viral infection. Together, our findings uncover that RuV encodes a VSR to evade antiviral RNAi response, which expands our understanding of RuV–host interaction and sheds light on the potential therapeutic target against RuV.

## 1. Introduction

RNAi is known as a conserved gene silencing mechanism that evolved as an important antiviral immune defense mechanism in diverse eukaryotes. In the RNAi-mediated antiviral pathway, virus-derived dsRNA (vi-dsRNA) produced from viral infection and replication can be sensed by the type III endoribonuclease Dicer and then cleaved into small viral interfering RNA (vsiRNAs) 21–24 nucleotides (nt) in length [1,2,3,4,5]. These active vsiRNAs are integrated into the host Argonaute (Ago) protein to form the RNA-induced silencing complex (RISC) with the help of other host factors. Subsequently, RISC containing vsiRNAs directs the degradation of cognate viral RNAs by the endonucleolytic activity in a sequence-specific manner [6,7].

In response, many viruses encode VSRs to counteract the antiviral RNAi pathway [8,9]. VSR proteins are widespread among RNA viruses that infect plants and animals and have also been identified in some DNA viruses [10,11,12]. In mammals, a number of mammalian virus-encoded VSRs, including Nodamura virus (NoV) B2 [13,14,15,16], influenza A virus (IAV) NS1 [17], enterovirus 3A [18,19], and dengue virus (DENV) NS2A [20] have been demonstrated to act as bona fide VSRs to antagonize antiviral RNAi in the context of authentic viral infections. Furthermore, hepatitis C virus (HCV) core and NS2 [21,22,23], human immunodeficiency virus (HIV) Tat [24], Ebola virus VP35 [25,26], severe acute respiratory syndrome coronavirus N protein [27,28], the yellow fever virus (YFV) capsid [29], and DENV NS4B [30] have been found to suppress the small hairpin RNA (shRNA)/siRNA-mediated RNAi in vitro. The phenomena of numerous viruses encoding VSRs to suppress the RNAi pathway highlight the importance of antagonizing antiviral RNAi during viral life cycles.

RuV is a genetically simple, enveloped, positive-strand RNA virus belonging to the family *Matonaviridae.* RuV is the sole member of the genus *Rubivirus* [31], while humans are the only natural hosts of RuV. Postnatal infections of RuV are usually mild, resulting in low-grade fever, scarlatiniform rash, or cervical lymphadenopathy [32]. However, maternal infection by RuV during pregnancy, particularly during the first trimester, can result in miscarriage and/or other congenital defects in newborns, collectively defined as congenital rubella syndrome (CRS) [33,34]. CRS is typified by a characteristic pattern of birth defects that include cataracts, deafness, blindness, cardiac defects, and intellectual disability [35]. Up to now, the epidemics of RuV infection occasionally occur in developing countries and CRS remains a major public health concern all over the world [36]. To date, there are no approved antiviral therapies specific for RuV.

The RuV genome consists of a single positive-strand RNA of approximately 12 kb, which encodes two nonstructural proteins—p150 and p90—and three major structural proteins—E1, E2, and capsid. The RuV capsid protein is the sole virus-encoded protein component of the nucleocapsid that participates in many processes critical for the viral life cycle, including membrane association, RNA-binding [37,38], modulating viral RNA replication [39,40], mitochondrial rearrangements [41], and inhibiting host protein translation by a nonenzymatic mechanism [42]. Our previous study demonstrated that the capsid protein encoded by the Semliki Forest virus (SFV), an important virus belonging to the *Alphavirus* genus in the family of *Togaviridae*, is a bona fide VSR that antagonizes antiviral RNAi in the context of SFV infection in cells [43]. Because the genomic structure and expression pattern of RuV and alphaviruses are similar, it would be intriguing to exploit whether the strategy of using capsid protein to suppress RNAi by SFV is also shared by RuV.

In this study, we identified that the RuV capsid could act as a potent VSR that inhibits Dicer-mediated siRNAs biogenesis by sequestrating dsRNAs and suppresses siRNA-induced RNAi. Moreover, we found that dimerization of the RuV capsid was required for its RNAi suppression activity. In addition, our data showed that the expression of the RuV capsid can rescue the replication of a VSR-defective model virus or replicon, implying that the RuV capsid can exert VSR function in a viral infection context.

## 2. Materials and Methods

### 2.1. Plasmids and RNAs

For the reversal-of-silencing assay in mammals, the plasmid pEGFP-C1 was used to express the EGFP protein. For the expression of the RuV capsid and its derivatives in mammalian cells, their ORFs were cloned into vector pRK-Flag. To express proteins in *Drosophila* S2 cells, the ORFs were constructed into the insect expression vector pAc5.1/V5-HisB, respectively. The full-length cDNA of the FHV RNA1 and FHV RNA1 ΔB2 was described previously [12]. For the purification of the MBP fusion capsid protein, its ORF was inserted into the pFastBac-MBP vector [43]. The EGFP-siRNA (siEGFP) was chemically synthesized by Rui Bo, Guangzhou, China.

### 2.2. Cell Culture and Transfection

HEK293T and Vero cells were maintained in Dulbecco’s modified Eagle medium (DMEM, Gibco) supplemented with 10% fetal bovine serum (FBS, Gibco, Grand Island, NY, USA), 100 U/mL penicillin, and 100 μg/mL streptomycin at 37 °C in an incubator with 5% CO_2_. The 293T-NoDice cell line was kindly provided by Bryan R. Cullen (Durham, NC, USA) and has been extensively used by us as previously described [18,20,43]. *Drosophila* S2 cells were cultured in Schneider insect medium (Gibco, Grand Island, NY, USA) with 10% FBS at 27 °C. Before transfection with FuGene HD reagent (Roche, Basel, Switzerland), the medium was changed to DMEM or Schneider insect medium containing 2% FBS without any antibiotic.

### 2.3. CRISPR/Cas9 Knockout

The Ago2 CRISPR/Cas9 KO Plasmid (sc-400813, Santa Cruz Biotech, Santa Cruz, CA, USA) was used to knockout the *ago2* gene in HEK293T cells according to the manufacturer’s protocol. Briefly, after transfection with the CRISPR/Cas9 KO plasmids, HEK293T cells were cultured for 3 days and then selected by puromycin (1 μg/mL) (Invivogen, San Diego, CA, USA). The sorting of the RFP-positive single cells was performed on a BD FACSAria III (BD Biosciences, San Diego, CA, USA) upon excitation with a 554 nm wavelength solid-state laser. After reaching a sustainable size (>5 × 10^2^ cells per clone), ∼10–200 cells of each clone were scraped off and collected using a 10 μL micropipette tip and screened for HDR-mediated repair of the mutation. The resulting clones were confirmed by DNA-sequencing and Western blotting.

### 2.4. Virus Infection

SFV (strain SFV4) and VSR-deficient SFV were from our laboratory; both were amplified in HEK293T cells [43]. EV-A71 (VR-1432, strain H) and VSR-deficient EV-A71 were from our laboratory; both were amplified in RD cells [18]. On the day of infection, the medium was changed with 2% FBS–DMEM, and then the viruses were added to HEK293T cells at an MOI of 1. Total RNAs were extracted at 12 and 24 h post infection (hpi) and subjected to qRT-PCR analysis.

### 2.5. Co-Immunoprecipitation

For immunoprecipitation, transfected HEK-293T cells were harvested in IP lysis buffer (20 mM Tris, 100 mM NaCl, 0.05% n-Dodecyl β-D-maltoside (Sigma-Aldrich, St. Louis, MO, USA), and protease inhibitor cocktail (Roche, Basel, Switzerland)), followed by immunoprecipitation using M2 Flag antibody (Sigma-Aldrich, St. Louis, MO, USA) or mouse IgG (Proteintech, Rosemont, IL, USA) and protein-A/G agarose beads (Roche, Basel, Switzerland) according to the manufacturer’s instructions. Briefly, cells were lysed at 4 °C for 30 min. Lysates were clarified at 12,000× *g* for 10 min at 4 °C and the lysates were pre-cleared by incubation with protein-A/G agarose beads (Roche, Basel, Switzerland) at 4 °C for 2 h. Then, the pre-cleared lysates were incubated with antibodies (anti-Flag or anti-IgG) together with protein-A/G agarose beads (Roche, Basel, Switzerland) at 4 °C for 12 h. The antibody-bound complexes were washed five times with the same lysis buffer. Finally, proteins were extracted from the complexes and analyzed by Western blotting.

### 2.6. Western Blotting and Chemical Compound

Cells were harvested in lysis buffer (50 mM Tris-HCl (pH 7.4), 150 mM NaCl, 1% NP-40, 0.25% deoxycholate, and a protease inhibitor cocktail (Roche, Basel, Switzerland)). The lysates were then subjected to 12% SDS-PAGE and Western blotting according to our standard procedures [18,44]. The antibodies used in this study included anti-tubulin (1:3000), anti-His (1:10,000), anti-Flag (1:5000), and anti-Dicer (1:2000), which were purchased from Proteintech, Rosemont, IL, USA. The JAK1/JAK2 inhibitor Ruxolitinib (INCB018424) was purchased from Selleck, Houston, TX, USA (CAS No. 941678-49-5).

### 2.7. Northern Blotting and qRT-PCR

Total RNAs were extracted using TRIzol reagent (Takara, Tokyo, Japan) according to the manufacturer’s instructions. For the detection of EGFP mRNA, 5 μg of total RNAs was subjected to denatured 1.5% agarose gels with 2.2 M formaldehyde. The separated RNAs were transferred onto the Hybond-A nylon membrane (GE Healthcare, Little Chalfont, Buckinghamshire, UK) and fixed at 120 °C for 15 min. The membranes were then hybridized with DIG-labeled probes in hybridization ovens at 65 °C overnight. The membranes were next incubated with anti-DIG antibody conjugated with alkaline phosphatase and exposed to a luminescent image analyzer LAS4000 (Fuji Film, Fuji, Japan). Probes for the detection of EGFP, Rp49, and GAPDH mRNA were complementary to nucleotide (nt) 520 to 700, nt 273 to 490, and nt 760 to 1060 of their ORF regions, respectively. The probe for the detection FHV RNA1 and subgenomic RNA3 was complementary to nt 2738 to 3058 of the B2 coding region. These probes were labeled with DIG-UTP (Roche, Basel, Switzerland) by in vitro transcription. For the detection of small RNAs, 20 μg of total RNAs was subjected to 7 M urea–15% PAGE and transferred to a Hybond-A nylon membrane (GE Healthcare, Little Chalfont, Buckinghamshire, UK). The membrane was chemically cross-linked in 1-ethly-3-(3-dimethylaminopropyl)-carbodiimide (EDC) at 60 °C. qRT-PCR using SYBR mix (Yeasen) [45] was carried out to detect the expression of EGFP mRNA, GAPDH mRNA, SFV NS1 mRNA, and EV-A71 mRNA.

### 2.8. RNA-Immunoprecipitation

RNA-IP was performed as previously described with minor modification [18]. Briefly, cells were lysed in a lysis buffer (20 mM Tris–HCl (pH 7.4), 200 mM NaCl, 2.5 mM MgCl_2_, 0.5% Triton X-100, 0.5 U/mL RNase inhibitor (Promega, Madison, WI, USA), and a protease inhibitor cocktail (Roche, Basel, Switzerland)) at 4 °C for 30 min. Lysates were clarified at 12,000× *g* for 10 min at 4 °C and the post nuclear lysates were pre-cleared by incubation with protein-A/G agarose beads (Roche, Basel, Switzerland) at 4 °C for 2 h. Then, the pre-cleared lysates were incubated with antibodies (anti-Flag or anti-IgG) together with protein-A/G agarose beads (Roche, Basel, Switzerland) at 4 °C for 12 h The antibody-bound complexes were washed five times with the same lysis buffer. Finally, proteins or RNAs were extracted from the complexes and subjected to Western blot or Northern blot analysis, respectively.

### 2.9. Expression and Purification of Capsid Protein

The coding regions of the RuV capsid protein were cloned into pFastBac-MBP. Sf9 cells were infected with the recombinant baculoviruses and harvested at 3 days post infection. Cell pellets were re-suspended, lysed by sonication, and subject to centrifugation for 30 min at 11,000× *g* to remove debris. To get rid of the possible contaminant co-purified from MBP–capsid via binding to RNA, the supernatant was treated with RNase A (Omega, Doraville, GA, USA) at a final concentration of 0.1 μg/μL for 4 h. All the purified proteins were concentrated using Amicon Ultra-30 filters (Millipore, Schwalbach, Germany). After that, the store buffer was exchanged to 50 mM 2-[4-(2-hydroxyethyl)-1-piperazinyl] ethanesulfonic acid (HEPES)–KOH (pH 8.0). All proteins were quantified by the Bradford method and stored at −80 °C in aliquots. Proteins were separated on 10% SDS-PAGE and visualized by Coomassie blue.

### 2.10. Gel Mobility Shift Assay

A gel mobility shift assay was performed in 50 mM HEPES–KOH (pH 8.0), 100 mM NaCl, 2 mM MgCl_2_, 1 mM tRNA, 2 mM DTT, and 20 U RNasin, at a total volume of 10 μL reaction with the indicated amount of proteins and 0.1 pmol double-strand RNA (dsRNA) or single-strand RNA (ssRNA). The dsRNA was labeled with DIG-UTP (Roche, Basel, Switzerland) by in vitro transcription and derived from 1 to 200 nt of EGFP. Reactions were incubated for 30 min at 25 °C. The reactions were terminated by the addition of 2.5 μL 5× sample buffer (20 mM Tris–HCl (pH 8.0), 30% glycerol and 0.1% bromophenol blue). The nucleic acid–protein complexes were separated by electrophoresis on 1.5% agarose gels and transferred to the Hybond-A nylon membrane (GE Healthcare, Little Chalfont, Buckinghamshire, UK). After that, the membrane was subjected to cross-linking at 120 °C and was incubated with anti-DIG-alkaline phosphatase antibody (Roche, Basel, Switzerland), followed by incubating with CDP-STAR (Roche, Basel, Switzerland) for 15 min at 37 °C. The signals were then detected by X-ray film (Fujifilm, Tokyo, Japan).

## 3. Results

### 3.1. RuV Capsid Is a Potent VSR in Mammalian Cells

**Figure 1:**
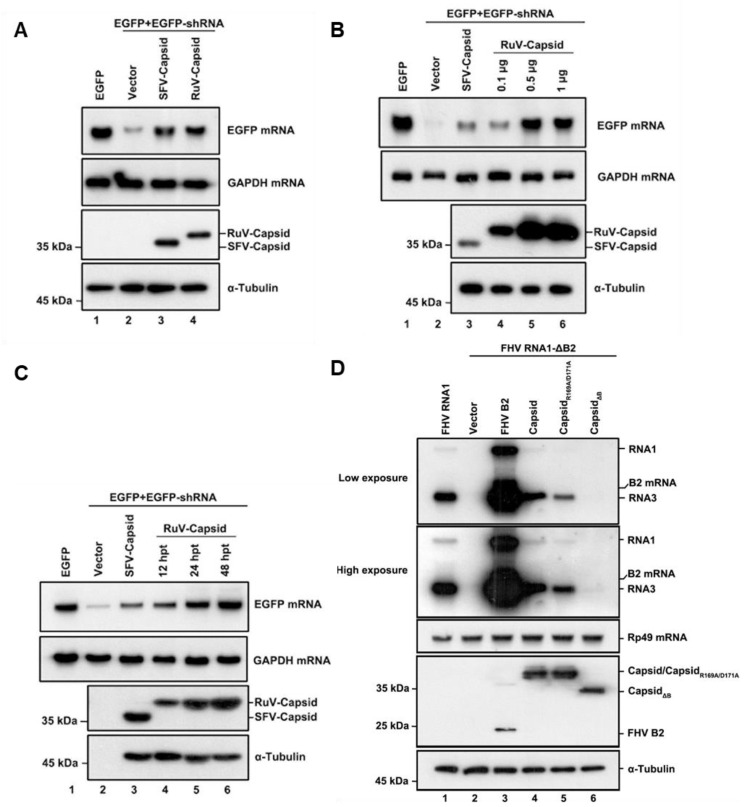
RuV capsid is a potent VSR in mammalian cells. (**A**) HEK293T cells were co-transfected with plasmids encoding EGFP (100 ng), EGFP-specific shRNA (shEGFP) (300 ng), and RuV capsid (1 μg), respectively. Cellular lysate and total RNAs were harvested and analyzed by Western and Northern blotting, respectively. Empty vector (Vec) and SFV capsid were used as a mock control and positive control, respectively. (**B**) Increasing amounts of RuV capsid-encoding plasmid were transfected into HEK293T cells in the reversal-of-silencing assay. At 48 h post transfection (hpt), Northern blotting was performed to determine the EGFP mRNA levels. (**C**) Northern blotting was performed 12, 24, and 48 hpt. (**D**) S2 cells were transfected with pMT-FHV RNA1 (FHV RNA1) (30 ng) or pMT-FHV-ΔB2 RNA1 (FR1 ΔB2) (600 ng), together with a plasmid encoding RuV capsid or FB2 (1 μg for each), as indicated. At 24 hpt, FHV RNA transcription was induced by incubation with CuSO_4_ (0.5 mM). At 24 h after induction, the total RNAs were harvested for Northern blot analysis. All the experiments have been independently repeated at least three times with reproducible results, and the representative results are shown.

To determine whether the RuV capsid protein acts as a VSR in vitro, we examined the capsid protein via a reversal-of-silencing assay as mentioned previously [18]. In this assay, HEK293T cells were co-transfected with the plasmids encoding EGFP and EGFP-specific shRNA (shEGFP), which is cleaved by Dicer to produce EGFP-specific siRNA and induces an RNAi response to degrade the EGFP transcript, together with a plasmid encoded Flag-tagged RuV capsid or SFV capsid, as a positive control. After 48 h transfection, EGFP mRNA levels were determined by Northern blotting, and the capsid proteins of RuV and SFV were determined by Western blotting. As shown in Figure 1A, ectopic expression of the RuV capsid efficiently restored the expression level of RNAi-silenced EGFP mRNA, as well as the SFV capsid did, indicating that the RuV capsid contains the VSR activity.

To investigate whether the VSR activity is dependent on protein expression levels, increasing amounts of RuV capsid were tested using the reversal-of-silencing assay (Figure 1B). The reversal effect of EGFP mRNA silencing increased progressively, along with the gradually increasing amounts of the transfected capsid-expressing plasmid (Figure 1B). We also detected EGFP mRNA at different time points, and the results showed that the reversal of EGFP mRNA silencing can be observed as early as 12 h after transfection of the capsid-expressing plasmid and was more effective at 24 and 48 h (Figure 1C).

To further confirm the VSR activity of the RuV capsid, we examined whether it can rescue the replication of a B2-deficient FHV RNA1 replicon (FHV-RNA1_ΔB2_) in *Drosophila* S2 cells [12]. This mutant FHV RNA1 replicon lost the ability to suppress RNAi, leading to defective self-replication of FHV RNA1 and subgenomic RNA3 in S2 cells (Figure 1D, compare lanes 1 and 2). Our data showed that the replication defect was partially rescued by the ectopic expression of either FHV B2 or the RuV capsid, respectively (Figure 1D, lanes 3 and 4). Taken together, our findings indicate that the RuV capsid exhibits in vitro VSR activity in cells.

### 3.2. RuV Capsid Inhibits Dicer-Mediated siRNA Generation and siRNA-Induced RNAi

**Figure 2:**
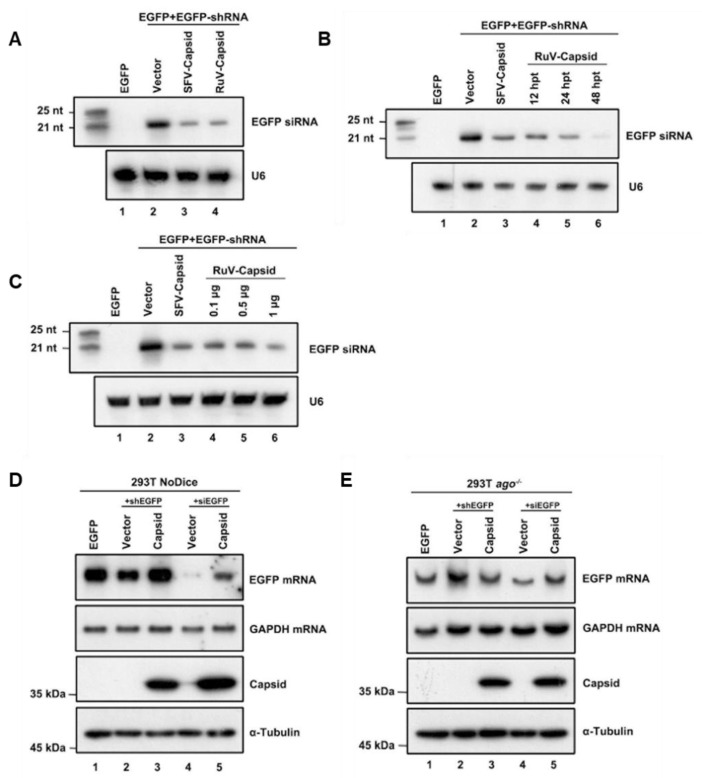
RuV capsid inhibits Dicer-mediated siRNA generation and siRNA-induced RNAi. (**A**) HEK293T cells were co-transfected with plasmids encoding EGFP (100 ng), EGFP-specific shRNA (shEGFP) (300 ng), and RuV capsid (1 μg), respectively. Small RNA Northern blotting was performed to examine the levels of EGFP-specific siRNA. U6 was used as a loading control. Empty vector (Vec) and SFV capsid protein were used as a mock control and a positive control, respectively. The synthetic 21- and 25-nt RNAs were used as size markers. (**B**) Increasing amounts of capsid-encoding plasmid were transfected into HEK293T cells in the reversal-of-silencing assay. At 48 hpt, small RNA Northern blotting was performed to examine the levels of EGFP-specific siRNA. (**C**) Northern blotting was performed 12, 24, and 48 hpt. (**D**,**E**) HEK293T NoDice (**D**) or *ago^−/−^* (**E**) cells were co-transfected plasmids encoding EGFP (100 ng), shEGFP (300 ng), or siEGFP (300 ng), together with the RuV capsid (1 μg), respectively. Total RNAs and proteins were harvested and analyzed by Northern and Western blotting, respectively. All the experiments have been independently repeated at least three times with reproducible results, and the representative results are shown.

After identifying that the RuV capsid contains in vitro VSR activity, we sought to examine the mechanism of how RuV capsid antagonizes RNAi. The shRNA-induced RNAi pathway requires Dicer-mediated dsRNA cleavage into siRNA. To determine whether the RuV capsid blocks this step, small RNAs harvested from HEK293T cells were subjected to Northern blotting with DIG-labeled probes targeting EGFP siRNA. In the presence of the RuV capsid, a reduction in 22-nt Dicer-processed siRNA was detected (Figure 2A, lane 4). Moreover, the production of 22-nt siRNA was gradually reduced along with the increasing protein expression levels of the RuV capsid (Figure 2B,C). This phenomenon was consistent with the EGFP mRNA levels observed in the reversal-of-silencing assays (Figure 1A–C).

In the RNAi pathway, when being processed from dsRNA, siRNAs are incorporated into RISC to mediate the cleavage of cognate RNAs [46]. Since we have found that the RuV capsid can inhibit shRNA-induced RNAi and Dicer-mediated siRNA production, it would be intriguing to examine whether the RuV capsid also suppressed siRNA-induced RNAi, which occurs after siRNA generation. To this end, Dicer- and AGO2-deficient HEK293T cells (NoDice and *ago2^−/−^* cells) were co-transfected with the plasmids encoding EGFP and shEGFP or chemically synthesized EGFP-specific siRNA (siEGFP), together with the plasmid for the RuV capsid, respectively. As shown in Figure 2D,E, the chemically synthesized siEGFP mediated the silencing of EGFP mRNA in NoDice rather than in *ago2^−/−^* cells, confirming that siEGFP can be efficiently incorporated into RISC to direct the cleavage of cognate mRNAs. Our findings showed that ectopic expression of the RuV capsid efficiently restored the levels of siRNA-silenced EGFP mRNA in NoDice cells, indicating that the RuV capsid can suppress siRNA-induced RNAi in mammalian cells.

### 3.3. RuV Capsid Suppressed Dicer-Mediated siRNA Production by Sequestrating dsRNA

**Figure 3:**
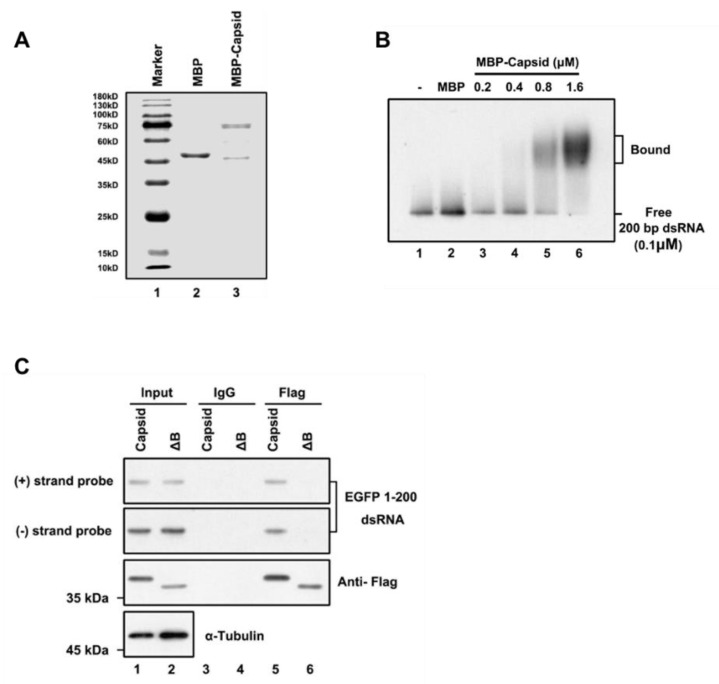
RuV capsid sequestrates dsRNA. (**A**) SDS-PAGE of purified recombinant RuV capsid. (**B**) Increasing amounts (0.2 to 1.6 μM) of MBP fusion capsid (MBP-capsid) and MBP protein as a negative control (2 μM) were incubated with 0.1 μM 200-nt DIG-labeled dsRNA at 25 °C for 30 min. Complexes were separated on 1.5% native-TBE agarose gel, transferred to membranes, and then incubated with anti-DIG antibody conjugated to alkaline phosphatase. (**C**) HEK293T cells were co-transfected with plasmids encoding RuV capsid_WT_ or capsid_ΔB_ (2 μg for each) and EGFP 1–200 nt dsRNA (1 μg), respectively. At 48 hpt, cell lysate was used to perform RNA-IP with anti-Flag antibody. The complex was harvested and analyzed by Northern and Western blotting, respectively. All the experiments have been independently repeated at least three times with reproducible results, and the representative results are shown.

To examine whether the RuV capsid inhibits siRNA production by directly binding to long dsRNA, we purified the recombinant maltose (MBP)-fusion capsid protein (MBP-capsid; Figure 3A, lane 3) and performed the electrophoretic mobility shift assay (EMSA) by incubating the in vitro-transcribed DIG-labeled 200-nt dsRNA together with the MBP-capsid. We found that the RuV capsid can directly interact with dsRNAs, and the shifting amount of labeled dsRNAs was increased along with the concentrations of MBP-capsid used in the reactions (Figure 3B).

Subsequently, we sought to examine whether the RuV capsid can associate with dsRNA in cells. For this purpose, we performed an RNA-IP assay in HEK293T cells ectopically expressing the Flag-tagged RuV capsid together with EGFP-specific dsRNA of 200nt in length. RNAs harvested from the Flag-precipitated complex were examined via Northern blotting with RNA probes that recognize positive- and negative-stranded EGFP sequences, respectively. We found that the RuV capsid indeed interacted with dsRNA (Figure 3C, lanes 5). Together, we conclude that the RuV capsid can suppress RNAi by sequestrating dsRNA from Dicer cleavage.

### 3.4. The Residues R169 and D171 of RuV Capsid Are Critical for RNAi Suppression Activity

After determining the in vitro VSR activity of the RuV capsid, we sought to identify the critical domains or amino acids required for its function. Previous studies show that the RuV capsid forms a disulfide-linked dimer [47]. Moreover, previous studies showed that homodimerization is critical for VSR’s activity [18,19,20,29,43]; we focused on the key region or residues responsible for the dimerization of the RuV capsid.

**Figure 4:**
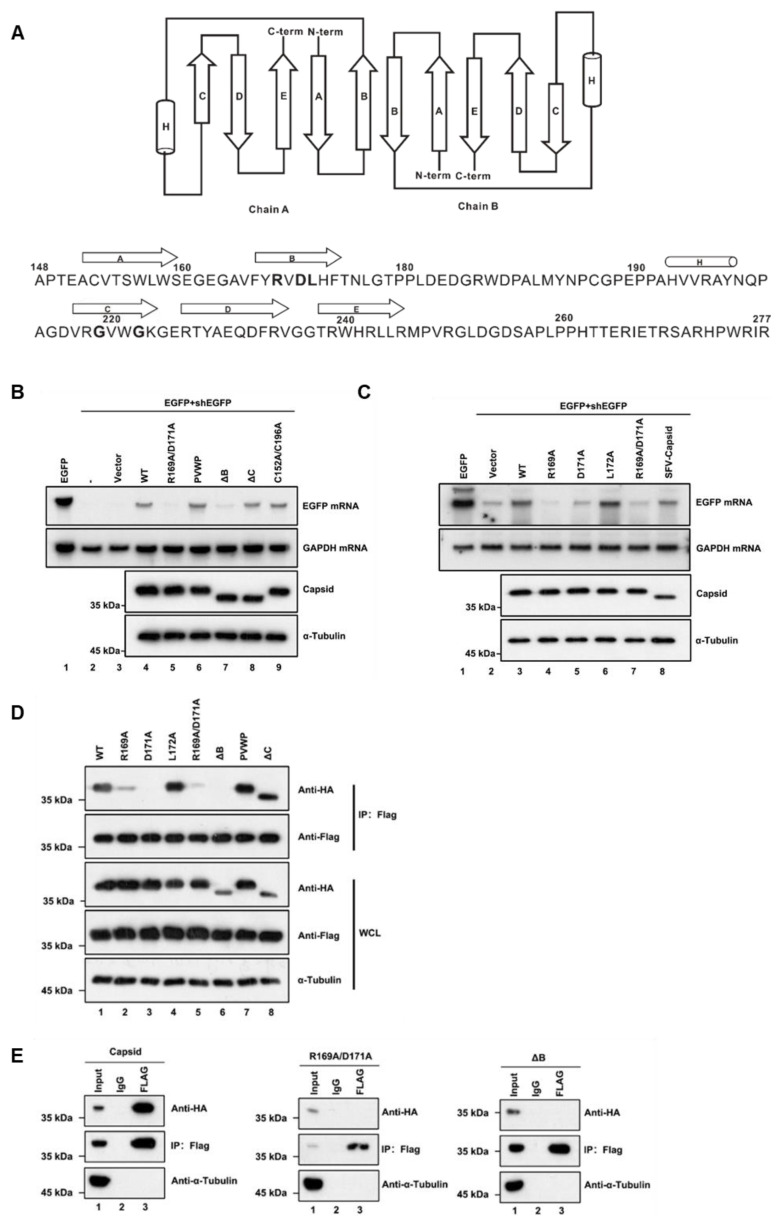
The residues R169 and D171 of the RuV capsid are critical for RNAi suppression activity. (**A**) Diagram of the RuV capsid dimer structure. The β-strands A–E were shown as arrows and the helix H was shown as a cylinder. (**B**,**C**) HEK293T cells were co-transfected with plasmids encoding EGFP (100 ng) and shEGFP (300 ng), together with a plasmid encoding RuV capsid_WT_, single-point or double-point mutations, or deletion mutants (1 μg for each). Empty vector (Vec) and SFV capsid were used as a mock control and a positive control, respectively. Cellular lysate and total RNAs were harvested and analyzed by Western and Northern blotting. (**D**) HEK293T cells were co-transfected with a plasmid encoding Flag-tagged RuV capsid_WT_ (1 μg), together with a plasmid encoding HA-tagged RuV capsid single-point or double-point mutations or deletion mutant (1 μg for each). At 48 hpt, cell lysate was used to perform co-IP with anti-Flag antibody. The complex was analyzed by Western blotting. (**E**) HEK293T cells were co-transfected with a plasmid encoding Flag-tagged and a plasmid encoding HA-tagged RuV capsid single-point or double-point mutations or deletion mutant (1 μg for each). At 48 hpt, cellular lysate was used to perform co-IP with anti-Flag antibody. IP samples were harvested and analyzed by Western blotting. All the experiments have been independently repeated at least three times with reproducible results, and the representative results are shown.

Each RuV capsid monomer consists of five antiparallel β-strands, including A–E, and a two-turn α-helix H between strands B and C (Figure 4A) [48]. To form a dimer, the residues of amino acids (aa) 169–175 of β-strand B from one monomer are hydrogen-bonded to the same but antiparallel β-strand B in the other monomer (Figure 4A). Moreover, the dimer can further form oligomerization through the interdimer molecular interface between capsid dimers, which involves β-strand C that is reported as the key residue essential for virus assembly and infection. In addition, Cysteine (C) at position 152 or 196 is reported to be important for the capsid’s dimer [48]. Accordingly, we constructed a set of capsid protein mutants and truncations, and examined their activities to suppress RNAi via the reversal-of-silencing assay in HEK293T cells. Our data showed that the region of β-strands B, ranging from aa 169 to 175, is critical for the VSR activity of the capsid (Figure 4B, lane 7, ΔB). The expression of capsid protein mutants and truncations was confirmed by Western blotting with anti-HA antibody (Figure 4B). On the other hand, either deleting the whole region of β-strand C or mutating the two key residues Glycine (G) to Prolines (P) within β-strand C showed no effect on the RNAi suppression activity of the RuV capsid (Figure 4B, lanes 6 and 8, PVWP and ΔC). The PVWP mutation was reported to disrupt the oligomerization of the RuV capsid but left its dimerization unaffected [48]. In addition, the C152A/C196A mutation did not affect the VSR activity (Figure 4B, lane 9).

To identify the critical residues within the region of aa 169 to 175 required for the VSR activity, we constructed several single-point or double-point mutations to alanine (A), and the resulting mutant capsid proteins were then examined via the reversal-of-silencing assay in HEK293T cells. The R169A, D171A, or R169A/D171A mutation (capsid_R169A_, capsid_D171A_, or capsid_R169A/D171A_) significantly suppressed the activity of the RuV capsid to inhibit RNAi (Figure 4C) in HEK293T cells. In addition, we found that the replication defect of FR1-ΔB2 cannot be rescued by the ectopic expression of either RuV capsid_R169A/D171A_ or capsid_ΔB_ (Figure 1D, lanes 5 and 6). As well, the truncation mutations (ΔB) abolished the dsRNA-binding activity of the RuV capsid examined by RNA-IP (Figure 3C, lane 6).

We sought to examine the relationship between the dimerization and the VSR activity of the RuV capsid. To this end, we performed co-IP assays with HEK293T cells ectopically expressing Flag-tagged capsid_WT_ together with HA-tagged capsid mutants. As shown in Figure 4D, the protein–protein interactions between capsid_WT_ and the mutant proteins (capsid_R169A_, capsid_D171A_, capsid_K169A/D171A_, and capsid_ΔB_) were remarkably attenuated. Moreover, the self-interaction activity of capsid_K169A/D171A_ or capsid_ΔB_ was completely blocked (Figure 4E).

Together, our findings indicate that dimerization of the RuV capsid is required for the VSR activity, and R169 and D171 residues are critical for both activities.

### 3.5. Ectopic Expression of RuV Capsid Rescue the Replication of VSR-Deficient SFV and Enterovirus A71 (EV-A71)

We sought to further investigate the role of the RuV capsid as a VSR during viral infection in vertebrate hosts. Previous studies showed that in differentiated somatic cells, the VSR activity during viral infection should be examined in VSR-disabled mutant viruses, which trigger an effective RNAi response [18]. Since the infectious DNA clone of RuV was unavailable to us, we used other VSR-deficient viruses including SFV_K124A/K128A_ and EV-A71_D23A_ instead in the subsequent tests. Our previous studies showed that the replication of these mutant viruses was restricted compared to that of wild type viruses due to loss of VSR activity, while either blocking the RNAi pathway or ectopic expressing the foreign VSRs can rescue the defective replication [18,43].

**Figure 5:**
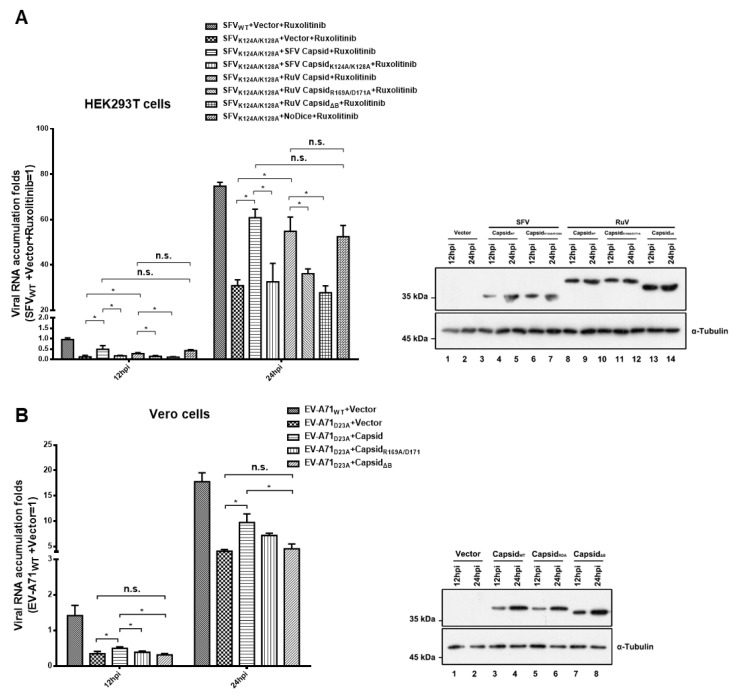
Ectopic expression of RuV capsid rescuing the replication of VSR-deficient SFV and EV-A71. (**A**) HEK293T or NoDice cells were transfected with either empty plasmid or a plasmid-encoded SFV capsid_WT_, capsid_K124A/K128A_, RuV capsid_WT_, capsid_R169A/D171A_, or capsid_ΔB_ as indicated. At 24 hpt, the cells were treated with ruxolitinib (10 μM) and then infected with SFV_WT_ or SFV_K124A/K128A_ at an MOI of 1. At 12 and 24 hpi, the levels of SFV genomic RNAs in cells were determined by qRT-PCR. (**B**) Vero cells were transfected with either empty plasmid or a plasmid-encoded SFV capsid_WT_, or capsid_K124A/K128A_ as indicated. At 24 hpt, the cells were infected with EV-A71_WT_ or EV-A71_D23A_ at an MOI of 1. At 12 and 24 hpi, the levels of EV-A71 genomic RNAs in cells were determined by qRT-PCR. Cellular lysate was harvested and analyzed by Western blotting. Empty vector was used as a mock control. All data represent the means and standard deviations of three independent experiments. * *p* < 0.05 as measured by two-way ANOVA, in GraphPad Prism. n.s., not significant.

HEK293T or HEK293T-NoDice cells treated with Ruxolitinib, a JAK1 and JAK3 inhibitor to exclude the potential interference of the interferon I system, were infected with SFV_WT_ or SFV_K124A/K128A_, and viral RNA accumulation was determined at 12 and 24 hpi, respectively. As expected, the viral RNA accumulation of SFV_K124A/K128A_ was lower than that of SFV_WT_ in HEK293T cells, while the genetic ablation of the RNAi pathway by Dicer knockout (NoDice) rescued the replication of SFV_K124A/K128A_ (Figure 5A). The viral RNA replication of SFV_K124A/K128A_ in HEK293T cells was increased by ectopic expression of RuV capsid_WT_ or SFV capsid_WT_, but not the VSR-deficient mutants of the RuV capsid (capsid_R1169A/D171A_ and capsid_ΔB_) or SFV capsid (capsid_K124A/K128A_) (Figure 5A). In addition, the viral RNA accumulation of SFV_K124A/K128A_ in cells expressing the RuV capsid was comparable to that in NoDice cells, indicating that the rescuing effect of the RuV capsid on the replication a VSR-deficient virus is dependent of the RNAi pathway.

The VSR activity of the RuV capsid was further examined in Vero cells infected with EV-A71_WT_ or EV-A71_D23A_. We found that the ectopic expression of RuV capsid_WT_, but not capsid_R1169A/D171A_ and capsid_ΔB_, still enhanced the viral RNA replication of EV-A71_D23A_ (Figure 5B). Taken together, our findings indicate that the VSR activity of RuV capsid is sufficient to rescue the replication of VSR-deficient virus.

## 4. Discussion

RuV is the etiological agent of a series of birth defects termed CRS that remains a global major public health concern, and better understanding of virus–host interactions would be helpful for identifying the pathogenesis of the viral disease and developing an antiviral therapy. RNAi is an antiviral response conserved in all eukaryotes, including mammals, and viruses encode VSRs to antagonize RNAi [49]. In this study, we found that the RuV-encoded structural protein capsid displayed VSR activity that suppresses Dicer-mediated siRNA production by sequestrating dsRNA and siRNA-induced RNAi in mammalian cells. Moreover, the VSR activity of the RuV capsid can rescue the defective replication of VSR-deficient viruses. Therefore, our findings support the notion that the RuV capsid can act as the VSR to facilitate viral replication by shielding viral dsRNA from Dicer cleavage and siRNA from RISC assembly.

Although belonging to different families, RuV and SFV share similar motif compositions, suggesting that their encoded proteins are likely to possess similar functions. Therefore, it is a rational prediction that the RuV capsid also contains VSR activity as does its counterpart, SFV. Moreover, our findings uncovered that the RuV capsid inhibits the RNAi pathway at two stages, including siRNA production and RISC assembly/function, which is consistent with the mode of action of the SFV capsid. Therefore, we speculate that using the capsid as VSR and targeting the steps of both siRNA production and RISC assembly/function are universal for multiple members in the family of *Togaviridae* to evade antiviral RNAi response.

Potent VSR activities were frequently observed in structural capsid proteins encoded by many viruses, such as the yellow fever virus capsid protein, SARS-CoV N, and SARS-CoV-2 N proteins. A virus-encoded capsid usually contains RNA-binding and dimerization activities, as association with viral genomic RNA, dimerization, and further oligomerization are necessary for viral nucleocapsid formation and the assembly of high-order structure of virions. In the case of RuV, our mutational analyses showed that mutation of R169A and D171A or deletion of the β-strand B region abolished the dimerization and dsRNA-binding of the RuV capsid, and, in turn, substantially reduced the VSR activity. Interestingly, the mutant PVWP, which was previously reported to disrupt the interaction between neighboring RuV capsid dimers to form high-order structure [48], have no influence on the VSR activity of the RuV capsid. These data indicate that dimerization, but not oligomerization, is required for the VSR activity of the RuV capsid, which is in line with previous findings that dimerization is a common feature for many VSRs, including NoV B2, EV-A71 3A, and SARS-CoV-2 N.

In summary, our work demonstrates that the RuV capsid functions as a VSR in facilitating viral replication by preventing dsRNA from Dicer cleavage and RISC assembly/function in mammalian cells. The activity of VSR is dependent on the dimerization of capsid, which possibly provides a possible direction for future anti-RuV drug exploitation. Moreover, it shows for the first time that RuV can antagonize antiviral RNAi, extending our knowledge about the interaction between *Rubivirus* and antiviral RNAi immunity.
